# Arboviral infections of the central nervous system: Mechanisms of invasion and long-term consequences

**DOI:** 10.1007/s13365-026-01328-4

**Published:** 2026-08-26

**Authors:** Olayinka M. Olajiga, Andrew G. MacLean, Brandon J. Beddingfield

**Affiliations:** 1 Division of Microbiology, Tulane National Biomedical Research Center, Covington, LA USA; 2 Department of Tropical Medicine, School of Public Health and Tropical Medicine, Tulane University, New Orleans, LA USA; 3 Division of Comparative Pathology, Tulane National Biomedical Research Center, Covington, LA USA; 4 Department of Microbiology and Immunology, Tulane University School of Medicine, New Orleans, LA USA; 5 Tulane Center for Aging, Tulane University School of Medicine, New Orleans, LA USA; 6 Louisiana Cancer Research Center, New Orleans, LA USA; 7 Tulane Cancer Research Center, New Orleans, LA USA; 8 Tulane Brain Institute, New Orleans, LA USA

## Abstract

Arboviruses are a diverse group of vector-borne viruses that cause significant global morbidity and mortality. Beyond classical systemic and febrile symptoms, many arboviruses can induce a spectrum of neurological complications, ranging from mild encephalopathy to severe neuroinvasive disease. This review summarizes current knowledge on major arboviruses from the Flaviviridae, Togaviridae, Peribunyaviridae, and Reoviridae families, emphasizing mechanisms of CNS invasion, neurological manifestations, and relevant case studies or outbreaks. Mechanisms of central nervous system (CNS) invasion are highlighted, including direct viral neurotropism, blood–brain barrier crossing such as Trojan horse–mediated leukocyte trafficking and immune-mediated neuropathology. Clinical syndromes, including encephalitis, myelitis, Guillain-Barré syndrome, and movement disorders, are discussed alongside relevant case studies and outbreak reports. This review provides an integrated perspective on arbovirus-induced neurological disease for clinicians, researchers, and public health professionals.

## Introduction

From acute outbreaks to chronic neurological sequelae and developmental impairments, the global health burden of neurotropic viruses is significant. Because of the great potential for infection and persistent illness in the CNS, these viruses account for a major source of morbidity and mortality across all age ranges. Neurotropic viruses are a diverse group of pathogens capable of infecting and replicating within cells of the nervous system, particularly the CNS. The history of scientific inquiry into arbovirus neurotropism viruses dates back to 1940, with a landmark study wherein a neurotropic virus was first isolated from the blood of a febrile Ugandan woman (Paul 1940). This virus, later named West Nile virus (WNV), was shown to cause encephalitis in Rhesus macaques and was found to be phylogenetically related to Japanese Encephalitis virus (JEV), both characterized by neuroinvasive and epidemic potential (Myint et al. 1999). This discovery paved the way to the understanding of viruses that invade the CNS and produce localized inflammation and systemic neurological illness. Neurotropic viruses induce a spectrum of neurological conditions, ranging from acute encephalitis to chronic neurodegenerative or autoimmune disorders, and in some cases, long-term neurological sequelae, developmental impairments, congenital abnormalities, as well as meningitis, myelitis, peripheral neuropathies, and seizure disorders (Myint et al. 1999).

Disease outcomes may be determined by the virus’s ability to enter the CNS, termed neuroinvasion, as well as the degree of CNS damage upon entry, termed neurovirulence. These factors are greatly influenced by viral characteristics, host immune responses, and host genetic susceptibility (Serafini et al. 2023). The CNS is protected by complex anatomical and physiological barriers, most notably the blood–brain barrier (BBB), which regulates molecular and cellular traffic between blood and brain parenchyma (Klein 2021). The BBB is formed by specialized endothelial cells, reinforced by astrocytic end-feet and pericytes, and maintains CNS homeostasis. Neurotropic viruses have evolved multiple strategies to circumvent or disrupt the BBB, including altering tight junction proteins, inducing endothelial apoptosis, crossing via transcytosis, or exploiting infected leukocytes in a “Trojan horse” mechanism and establish infection in neurons, glia, or other CNS structures (Ampie and McGavern 2022). Some viruses also bypass the BBB entirely by retrograde transport along peripheral nerves, demonstrating the versatility of viral entry strategies (Kaya and Ahishali 2021).

The global burden of Arthropod-borne viruses, or arboviruses, is substantial, often leaving survivors with lasting impairments, including cognitive deficits, motor dysfunction, or congenital neurological abnormalities. Though Dengue virus is mostly associated with hemorrhagic symptoms and fever-inducing diseases, it also displays neurotropic characteristics with neurological symptoms including encephalitis, myelitis, and Guillain-Barré syndrome. These impacts strongly support intensified vaccination efforts, comprehensive surveillance, and sustained public health interventions, especially in regions of high prevalence and resource limitations.

## Arthropod-borne viruses

The families of arboviruses with significant neurovirulent potential span multiple families, each invading the CNS via various mechanisms and exhibiting differing pathology.

### Family Flaviviridae

#### West Nile virus

##### Mechanisms of WNV CNS invasion

**Fig. 1:**
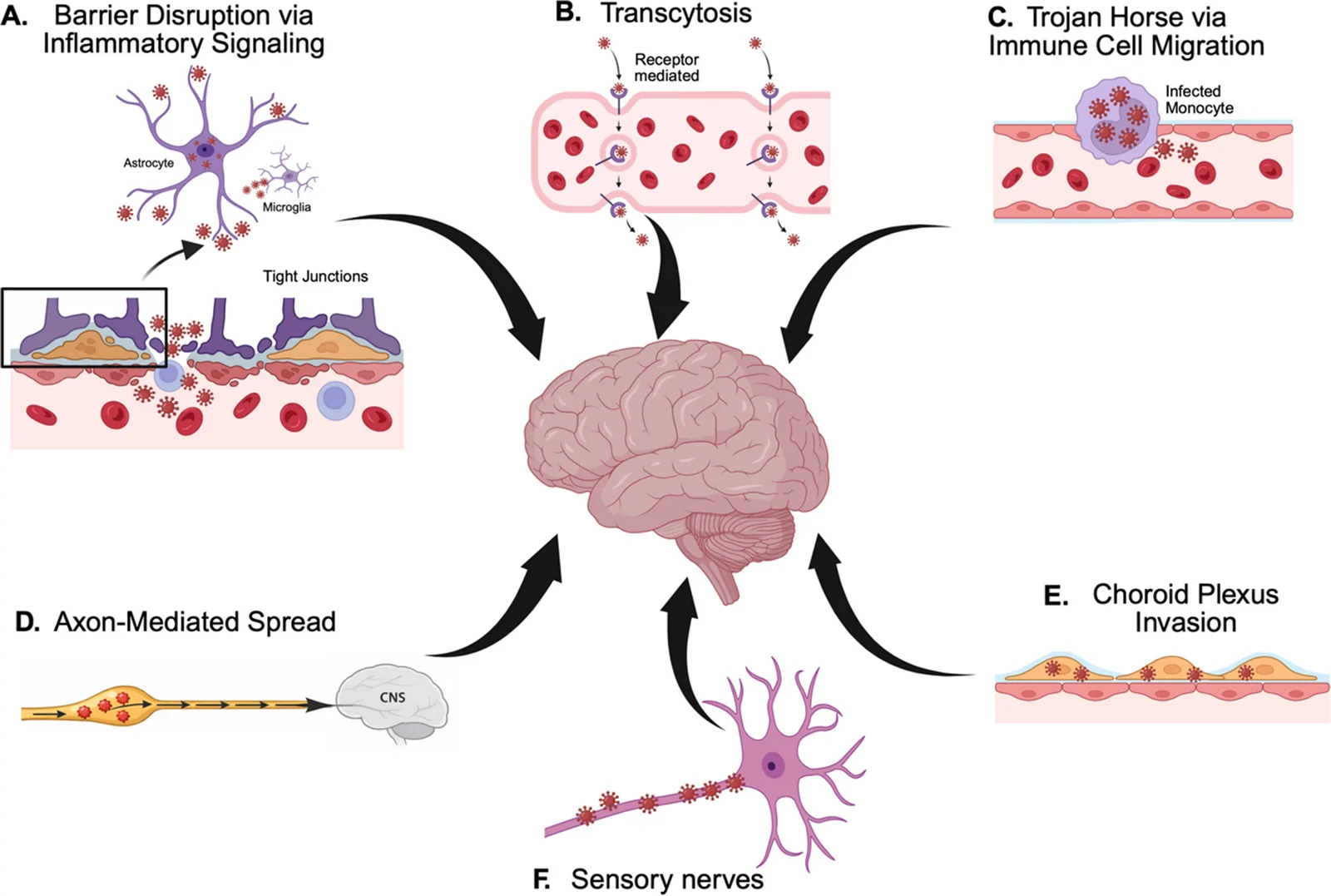
Mechanisms of Arbovirus invasion of CNS. Following initial replication in peripheral tissues, viruses access the central nervous system through multiple, non-mutually exclusive pathways: (**A**) Barrier disruption, involving direct infection of endothelial cells or inflammation-induced compromise of barrier integrity; (**B**) Transcytosis via receptor present on endothelial cells; (**C**) Trojan horse mechanism, in which infected immune cells traffic across protective barriers; (**D**) Axon-mediated spread, via retrograde transport along neurons, allowing direct CNS access; (**E**) Entry via the choroid plexus, facilitating passage into cerebrospinal fluid and widespread access to the CNS; and (**F**) Sensory neuron invasion, allowing direct access from peripheral sensory structures such as the olfactory pathway; (**G**) Vascular route, where circulating virus crosses the BBB or blood–CSF barriers. These pathways contribute to CNS dissemination and the development of neurologic manifestations

West Nile virus is transmitted primarily by Culex mosquitoes, with birds as reservoirs (Ciota 2017). Human cases are most prevalent in Africa, Europe, the Middle East, and the Americas, especially the U.S., where the virus is now endemic and exhibits seasonal transmission driven by fluctuations in mosquito vector abundance (Brussow and Figuerola 2025; D’Amore et al. 2022). After a mosquito bite, WNV replicates in peripheral tissues, including endothelial cells (Fig. 1A – barrier disruption), and circulates in the bloodstream, resulting in systemic dissemination reaching the CNS (Mustafa et al. 2019). To cross the BBB, WNV targets brain microvascular endothelial cells (BMECs), which form the brain capillaries (Marshall et al. 2024). Unlike cytolytic viral infections that cause gross tissue damage, WNV establishes a non-cytopathic infection in BMECs, allowing the virus to persist and manipulate the host cell’s machinery without triggering early immune alarms or immediate cell death (Mustafa et al. 2019). During WNV encephalitis (Roe et al. 2012), WNV manipulates intracellular trafficking pathways to weaken the BBB by activating a dynamin-dependent endocytosis pathway, leading to downregulation of claudin‑1, junctional adhesion molecule‑1 (JAM‑1), Zonula occludens‑1 (ZO‑1), occludin, vascular endothelial (VE)‑cadherin, and β-catenin, along with upregulation of matrix metalloproteinases (MMP‑1, ‑3, ‑9) making the barrier more permeable. The consequence of this tight junction degradation is an increase in paracellular permeability, which facilitates the translocation of virus particles, immune cells, or inflammatory mediators into the CNS, contributing to neuropathogenesis (Mustafa et al. 2019; Al-Obaidi et al. 2018). WNV also crosses the BBB via transcytosis (Fig. 1B), a process in which virus envelope proteins interact with specific endothelial cell receptors such as DC-SIGN/DC-SIGNR, HSPGs, and TIM/TAM receptors, gets internalized at the apical surface through clathrin-mediated endocytosis, trafficked across by intracellular vesicles, and released basolaterally without disrupting tight junction integrity (Mustafa et al. 2019; Marshall et al. 2024; Roe et al. 2012).

WNV also exploits a Trojan horse mechanism (Fig. 1C), in which circulating leukocytes in the periphery become infected and cross the BBB during normal immune surveillance. This process is facilitated by increased expression of endothelial adhesion molecules, ICAM-1 and VCAM-1, on BMECs, which promotes adhesion and transendothelial migration of infected cells into the brain parenchyma (Mustafa et al. 2019). In parallel, WNV activates astrocytes and microglia, which secrete inflammatory cytokines and chemokines that recruit additional peripheral immune cells into the CNS, some of which become infected and subsequently act as Trojan horses (Mustafa et al. 2019).

In addition to host factors, viral proteins also directly impact BBB integrity. Non-structural protein 1 (NS1), secreted by WNV during infection, has been implicated in endothelial dysfunction by targeting the endothelial glycoclayx (Lo et al. 2022), the carbohydrate-rich layer lining the luminal surface of blood vessels that protects endothelial cells. NS1-mediated degradation of the glycocalyx compromises this protective barrier, leading to increased vascular leak and heightened BBB permeability, exacerbates the inflammatory milieu and promotes viral dissemination within the CNS (Puerta-Guardo et al. 2016). Collectively, WNV employs a multi-pronged strategy to invade the CNS, combining mechanical disruption of the BBB, hijacking of host cellular pathways, immune cell-mediated trafficking, and viral protein-induced endothelial dysfunction.

##### Neurological manifestations of WNV

WNV infection ranges from asymptomatic to severe neuroinvasive disease, termed West Nile neuroinvasive disease (WNND). While an estimated 80% of WNV infections are subclinical and likely underreported, approximately 20% of infected individuals develop West Nile fever, characterized by fever, headache, myalgia, arthralgia, rash, and gastrointestinal symptoms that typically resolve within one week (Petersen et al. 2013). Less than 1% of the West Nile fever patients progress to WNND, which encompasses meningitis, encephalitis, acute flaccid paralysis (AFP), and movement disorders, with a case fatality rate of approximately 10%. WNV encephalitis commonly presents with fever and altered mental status ranging from confusion to coma and can include seizures, tremor, and extrapyramidal features such as rigidity and bradykinesia (Sejvar et al. 2003). A prospective study of 16 patients with serologically confirmed WNV infection reported that tremor was observed in 94%, myoclonus in 31%, and Parkinsonian features such as gait instability and cognitive slowing in 69% of the cases (Sejvar et al. 2003). These movement abnormalities often reflect involvement of deep gray matter structures, including the basal ganglia and thalamus, and may persist long after the acute illness (Lenka et al. 2019). Less frequent findings include ataxia, chorea, and opsoclonus-myoclonus syndrome (Cooper and Said 2014). Meningitis typically presents with headache, neck stiffness, photophobia, and lymphocytic pleocytosis. In contrast, WNV-associated AFP mimics poliomyelitis, with asymmetric limb weakness developing rapidly and often without sensory loss due to anterior horn cell involvement (Sejvar et al. 2010). Respiratory muscle paralysis may require mechanical ventilation, and while one-third of patients recover near baseline, others have lasting deficits. Other neurologic manifestations include cranial neuropathies, seizures, optic neuritis, transverse myelitis, Guillain-Barré–like syndromes, and autonomic disturbances such as bladder dysfunction or cardiac arrhythmias (Yu et al. 2020). Among survivors of acute illness, long-term sequelae are common, especially in neuroinvasive cases. Studies estimate that 40–80% of patients experience persistent fatigue, memory impairment, tremor, or weakness for months to years (Lenka et al. 2019; Sejvar et al. 2010). In one Canadian cohort, nearly half of WNND survivors still reported cognitive fatigue and motor symptoms two years post-infection (Samaan et al. 2016).

**Case studies/outbreaks of WNV**

**Table:** 

Year	Location	Reported cases	Neuroinvasive cases	Deaths	Notable features	References
1996	Bucharest, Romania	393	352	Not specified	Encephalitis, meningitis	Tsai et al. 1998
1999	New York, USA	62	Not specified	7	Encephalitis, and AFP	Nash et al. 2001; Lanciotti et al. 1999
2000	Israel	417	326	35	Encephalitis and meningitis	
2002	USA	4,000 +	1 in 150	284	Meningitis, encephalitis, parkinsonism	Petersen et al. 2013; Sejvar et al. 2003
2003	Colorado, USA	2,947	621	63	Meningitis, encephalitis, and AFP	CDC 2025
2012	Texas, USA	1,875	305	89	Meningioencephalitis, cognitive dysfunction,	Yango et al. 2014; Murray et al. 2013
2012	Ontario, Canada	1,884	345	1 out of 7 patients	Encephalitis, meningitis, meningomyelitis, and Cognitive dysfunction,	Jiao and Main 2014
2010–2017	Greece	672	474	81	Encephalitis, cranial neuropathies	Pervanidou et al. 2014, 2020
2013	Serbia	~ 302	32	Not specified	Meningitis, consciousness disorder	Sevic et al. 2015
2018	Europe (18 countries)	1,993	1,389	202	Encephalitis, meningoencephalitis or meningitis	Young et al. 2021
2018	USA	2,647	1,658	165	Meningitis, encephalitis, and acute flaccid paralysis	McDonald et al. 2019
2012–2020	Italy	1145	487	11	Encephalitis	Ricco et al. 2021
2020	Andalusia, Spain	71	All	8	Meningoencephalitis	Figuerola et al. 2022
2024	Israel	125	64	12	Meningoencephalitis and disability accrual	Mor et al. 2024

#### Zika Virus

##### Mechanisms of ZIKV CNS invasion

Zika virus is transmitted mainly by the mosquito species *Aedes aegypti* and *Aedes albopictus* (Gregory et al. 2017). Unlike WNV, it also spreads sexually and vertically from mother to fetus during pregnancy (Zamani and Zamani 2017; Marban-Castro et al. 2021). Transmission via blood transfusion has been documented during outbreaks, and rare cases of laboratory exposure has been reported (Gimenez-Richarte et al. 2022; Hills et al. 2021). Transmission is most prevalent in tropical and subtropical regions of the Americas, Africa, and Asia, particularly where *Aedes* mosquitoes are prevalent (Guo et al. 2022). ZIKV shares several mechanisms of CNS entry with other neurotropic flaviviruses, including infection of endothelial cells and trafficking via immune or neural routes. Following peripheral replication and viremia, ZIKV infiltrates circulating leukocytes, including monocytes and macrophages, using them as Trojan horses to cross into the BBB and CNS parenchyma during immune cell recruitment into the CNS (Fig. 1C) (Cle et al. 2020). ZIKV also targets BMECs, modulating the expression and localization of tight junction proteins essential for BBB integrity, similar to WNV without causing widespread cell death or junctional degradation. These infected cells then remain viable and release virus basolaterally into the CNS, suggesting transcytosis or receptor-mediated endocytosis as early modes of entry (Papa et al. 2017; Mladinich et al. 2017).

ZIKV also elicits a robust inflammatory response that significantly contributes to BBB disruption and CNS invasion. Infected endothelial cells and CNS-resident cells release cytokines such as IL-6 and TNF-α, which promote BBB disruption, along with IFN-β, which alters endothelial signaling and tight junction regulation (Fig. 1A), collectively facilitating viral passage into the CNS and immune cell infiltration (Papa et al. 2017). ZIKV activates key inflammatory signaling pathways, including NF-κB and MAPK, within endothelial and glial cells, leading to upregulation of adhesion molecules such as ICAM-1, VCAM-1 and proinflammatory mediators that perpetuate endothelial activation, barrier dysfunction and leukocyte transmigration (Cle et al. 2020). This sustained inflammatory milieu promotes immune cell infiltration, microglial activation, and subsequent neuronal injury.

In addition to hematogenous spread, ZIKV also invades the CNS via neural routes (Fig. 1D). ZIKV has been shown in murine and primate models to infect peripheral sensory neurons, including those in the olfactory epithelium, and, after retrograde transport along axons, spreads within the brain (Ozdener et al. 2020; Oh et al. 2017). The detection of ZIKV RNA and infectious particles in the olfactory bulb shortly after inoculation, prior to detectable viremia, supports axon-mediated CNS entry (Ozdener et al. 2020). ZIKV also targets the blood cerebrospinal fluid barrier (BCSFB) through interactions with receptor tyrosine kinases such as AXL which is highly expressed on immature or activated pericytes of the choroid plexus (Fig. 1E). Viral engagement of AXL facilitates entry into pericytes and subsequent disruption of the BCSFB, granting access to the cerebrospinal fluid and deeper brain regions (Kim et al. 2020). AXL expression on astrocytes and microglia also enables viral propagation within the CNS (Fig. 1A) where ZIKV exploits AXL to suppress antiviral immune responses by upregulating SOCS1 and SOCS3, which inhibit JAK/STAT signaling and blunt type I interferon responses (Chen et al. 2018). This immune evasion strategy allows ZIKV to replicate in myeloid cells without triggering apoptosis or strong inflammatory responses, preserving their migratory capacity and promoting viral dissemination. Although AXL is the most well-characterized entry receptor, other members of the TAM (Tyro3, AXL, Mer) and TIM families also contribute to ZIKV internalization via receptor-mediated endocytosis (Yu et al. 2025).

##### Neurological manifestations of ZIKV

ZIKV's most devastating impact, termed congenital Zika syndrome (CZS), occurs when the infection is transmitted vertically during pregnancy. Affected offspring commonly present with microcephaly, which is associated with a spectrum of abnormalities including calcifications at the cortical–subcortical junction, ventriculomegaly, brainstem and cerebellar hypoplasia, and lissencephaly, consistent with imaging findings of cortical malformations, and white matter loss^,^ (Wu et al. 2018). Beyond structural anomalies, affected infants frequently develop seizures, developmental delays, motor deficits, and visual abnormalities such as optic nerve disease or retinal changes. During the 2013–14 Polynesian and 2015–16 Latin American outbreaks, ZIKV infection was linked to a significant rise in acute demyelinating neuropathies, particularly Guillain-Barré Syndrome (GBS). Case series report that GBS constituted nearly half of adult admissions associated with neurological impairment from ZIV infection (neuroZika), frequently with respiratory compromise requiring intensive care support (Lannuzel et al. 2019). Interestingly, the temporal onset of neurological symptoms frequently occurs during the acute infectious phase, occasionally overlapping with febrile illness (Munoz et al. 2017), which suggests direct viral neurotropism in addition to an autoimmune mechanism. Clinical presentations vary from classical ascending paralysis to cranial neuropathies, sensory disturbances, and autonomic dysfunction (Lannuzel et al. 2019). Although less common than GBS, ZIKV can cause encephalitis, meningoencephalitis, myelitis, and acute disseminated encephalomyelitis. MRI findings in severe adult cases demonstrate subcortical T2/FLAIR hyperintensities, brainstem involvement, and cauda equina root enhancement. Weakness, cranial nerve palsies, seizures, and altered mental status are often present from reports in ICU settings with up to 20% of hospitalized confirmed neuroZika patients exhibiting mixed central and peripheral syndromes, with a mortality rate of ~ 2% in ZIKV-ICU patients (Lannuzel et al. 2019; Sebastian et al. 2017). Persistent neurological deficits are common following ZIKV–associated neuroinvasive disease, including cognitive impairment and motor weakness (Lannuzel et al. 2019). Congenital cases also endure lifelong challenges requiring comprehensive clinical and developmental support for neurodevelopmental delay, epilepsy, sensory disabilities, and growth defects such as intrauterine growth restriction and postnatal failure to thrive. Additional neurological features have emerged including transverse myelitis and encephalomyelitis, optic neuritis and other cranial neuropathies, and stroke-like syndromes in adults (Munoz et al. 2017).

**Case studies/outbreaks of ZIKV**

**Table:** 

Year(s)	Location	Reported cases	Neuroinvasive cases	Deaths	Notable features	References
2007	Yap Island, Micronesia	~ 185 suspected; 49 confirmed	None reported	0	First documented ZIKV outbreak in humans; no neurological symptoms reported	Duffy et al. 2009
2013–2014	French Polynesia	~ 8,723 suspected; est. 30,000 total infected	61	0	First strong link to GBS during ZIKV outbreak	Kucharski et al. 2016; Watrin et al. 2016; Besnard et al. 2016
2015–2016	Brazil	~ 1.5 million estimated	8,301	76	Emergence of CZS; severe spike in microcephaly and GBS	Heukelbach et al. 2016; França et al. 2018; Magalhães-Barbosa et al. 2016
2015–2016	Colombia	108,000	1,163	Not specified	Confirmed ZIKV–microcephaly link; GBS	Mendez et al. 2017; Charniga et al. 2021
2015–2016	El Salvador	73,395	14.7 cases per 100,000 population per year	Not specified	Sudden rise in GBS cases concurrent with ZIKV outbreak	Shutt et al. 2017
2015–2016	Haiti	3,036	42	Not specified	Case reports of GBS and congenital abnormalities	Journel et al. 2017
2016	Puerto Rico	39,717	1%	13	Surveillance confirmed ZIKV–GBS link; rise in congenital Zika syndrome	Sharp et al. 2020
2016–2017	Mainland, USA	5,168; 224 locals	Not reported	1	GBS and congenital Zika syndrome in several states, especially Florida	Hall et al. 2018; William et al. 2016
2016–2017	Dominican Republic	5,226	288	17	Increased incidence of GBS reported by national health authorities	Pena et al. 2019
2016	Cape Verde	~ 7,500 suspected	18	Not specified	First ZIKV outbreak in Africa with documented microcephaly	Lourenco et al. 2018; Faye et al. 2020

#### Japanese encephalitis virus

##### Mechanisms of JEV CNS invasion

Japanese encephalitis virus is transmitted to humans through the bite of an infected mosquito, primarily the *Culex tritaeniorhynchus* mosquito. It is a major cause of viral encephalitis in Asia and the Western Pacific including countries such as India, China, Japan, South Korea, Thailand, Vietnam, Cambodia, Australia, and Indonesia (Wang and Liang 2015). The virus was first isolated in 1935 from the brain of a fatal encephalitis case in Japan (Solomon et al. 2000). Following infection, JEV replicates in dendritic cells and keratinocytes, before disseminating through the lymphatic system (Kumar et al. 2022; Sharma et al. 2023). Once viremia is established, the virus accesses the CNS through the hematogenous, Trojan horse, and neuronal routes (Fig. 1A, C, D, F) (Kumar et al. 2022). The Trojan horse mechanism is the term utilized when infiltrating immune cells such as monocytes and macrophages become infected and transport the virus across the BBB, delivering it directly into the brain parenchyma (Hsieh and St John 2020). These mechanisms enable JEV to evade some of the early innate immune defenses and facilitates widespread dissemination within neural tissues.

Another mechanism involves direct infection of BMECs (Sharma et al. 2023). JEV infection induces alterations in tight junction proteins such as claudin-5, occludin, and ZO-1 that are essential for maintaining BBB integrity, leading to increased paracellular leakage between endothelial cells and allowing viral particles to traverse the barrier (Li et al. 2015). In parallel, the virus stimulates the release of MMP-2 and MMP-9, which degrade extracellular matrix components and exacerbate endothelial dysfunction. This process is amplified by the robust proinflammatory response JEV elicits from both peripheral immune cells and resident CNS cells (Yadav et al. 2022). Infected BMECs, astrocytes, and microglia secrete proinflammatory cytokines, including TNF-α and IL-6, as well as chemokines such as CCL2 and CXCL10, which promote leukocyte adhesion and transmigration (Kumar et al. 2022; Yadav et al. 2022). These recruited leukocytes secrete IFN-γ, further amplifying endothelial activation and adhesion molecule expression at the BBB. This inflammatory milieu not only compromises BBB integrity but also facilitates the infiltration of immune cells into the CNS (Kumar et al. 2022; Yadav et al. 2022).

In addition to hematogenous routes, JEV also reaches the CNS via neuronal transport (Fig. 1D) (Hsieh and St John 2020). Experimental models have demonstrated that JEV can invade the brain through peripheral nerves, particularly the olfactory and trigeminal pathways (Fu et al. 2019; Ricklin et al. 2016). These nerves provide direct anatomical connections between peripheral sites of replication and the brain, bypassing the BBB altogether (Fig. 1F). Following peripheral infection, viral RNA and antigens have been detected in the olfactory bulbs and brainstem, suggesting retrograde axonal transport as a viable route of neuroinvasion (Hsieh and St John 2020). This neural entry of JEV may be particularly relevant in pediatric cases as the developing CNS provides a more permissive environment for viral replication. Studies in developing cortical organoids show that neuronal immaturity, weaker antiviral responses, and the abundance of neural progenitor cells increase susceptibility to JEV infection, contributing to the heightened vulnerability of infants and young children (Zhang et al. 2018).

JEV’s ability to infect a wide range of CNS-resident cells including neurons, astrocytes, microglia, and BMECs further facilitates its persistence and pathogenesis (Hsieh and St John 2020). Once in the CNS, JEV induces apoptosis in infected neurons, activates microglia, and promotes astrogliosis, leading to neuronal dysfunction and long-term sequelae in survivors (Sharma et al. 2023; Yadav et al. 2022). The engagement of specific host receptors is believed to play a role in viral tropism and cellular entry. Although definitive CNS entry receptors for JEV remain under investigation, several molecules have been implicated in viral attachment and internalization, including DC-SIGN, AXL, TAM receptors, laminin receptors, and specific heat shock proteins (Hsp70 and Hsp90) (Filgueira and Lannes 2019). These interactions facilitate receptor-mediated endocytosis and intracellular trafficking that support productive infection.

##### Neurological manifestations of JEV

Clinical manifestations of JEV-associated neuroinvasive disease span a broad spectrum, from mild febrile illness to encephalitis, with high morbidity and mortality rates. Mortality in JEV-infected individuals ranges from 20–30%, and among survivors, 30–50% experience long-term neurological deficits (Cheng et al. 2022). The hallmark presentation of symptomatic JEV infection is acute encephalitis, characterized by fever, altered mental status, and seizures (Raman Sharma et al. 2025). Encephalopathy may progress rapidly, often accompanied by neck stiffness, photophobia, and coma (Misra and Kalita 2010). In children, seizures and movement disorders, such as choreoathetosis, dystonia, or parkinsonian features are observed (Dale and Mohammad 2024). Extrapyramidal signs and symptoms are a distinctive and frequently reported manifestation of JEV infection, resulting from viral infection of the thalamus, basal ganglia, brainstem, and spinal cord, as demonstrated in both imaging and autopsy studies (Raman Sharma et al. 2025). Movement disorders are more prevalent in pediatric patients, correlating with damage to the basal ganglia and thalami visible on MRI. Seizures are also common features of JEV in pediatrics with some developing status epilepticus. AFP, resembling poliomyelitis, is another recognized but less frequent manifestation, with viral RNA detected in the cerebrospinal fluid, indicating spinal cord involvement (Ghosh et al. 2020). It typically results from anterior horn cell damage leading to asymmetric limb weakness or monoplegia, sometimes accompanied by cranial nerve palsies, with recovery being variable and often incomplete. Neuropsychiatric complications may emerge during recovery, including irritability, agitation, psychosis, or cognitive impairment (Kar et al. 2025). Long-term follow-up study indicates that up to 30–50% of JEV survivors experience persistent neurological or psychiatric sequelae, such as cognitive impairments, behavioral issues, post-encephalitic epilepsy, and poor scholastic performance (Singhai and Nishant 2022). Neuroimaging in JE commonly reveals bilateral thalamic lesions, often hyperintense on T2-weighted MRI, and in some cases, involvement of the basal ganglia, midbrain, cerebellum, and spinal cord (Ramli and Bae 2023; Phukan et al. 2021). These radiological findings correlate closely with clinical symptoms and can help distinguish JEV from other causes of viral encephalitis (Misra and Kalita 2010).

**Case studies/outbreaks of JEV**

**Table:** 

Year(s)	Location	Reported cases	Neuroinvasive cases	Deaths	Notable features	References
1924	Japan	> 6000	Not separately specified	50%	One of the earliest large-scale outbreaks; referred to as “summer encephalitis”	Hiroyama 1962; Solomon et al. 2000
1990s–2000s	Vietnam	> 1,500 annually	Not specified	Not specified	Significant JE activity	Yen et al. 2010
2005	Gorakhpur, Uttar Pradesh, India	~ 5,700	Not separately specified	1344	Major pediatric outbreak, bilateral thalamic lesions confirmed by MRI and autopsy	Parida et al. 2006
2006	Shanxi Province, China	66	Not specified	19	Outbreak prior to expanded JE vaccine coverage	Wang et al. 2007
2022	Assam	434	Not separately specified	93	Vision problems, hearing difficulties, and neck stiffness	Kumari et al. 2022
2022	Australia	42	Not separately specified	7	Majority of cases are occurring around the affected piggeries particularly in the older age group	Hutchinson et al. 2022; Mackenzie and Smith 2024

#### Tick-borne encephalitis virus

##### Mechanisms of TBEV CNS invasion

Tick-borne encephalitis virus is primarily transmitted through the bite of infected *Ixodes* ticks, particularly *I. ricinus* and *I. persulcatus* (Pustijanac et al. 2023). Infection can also occur via consumption of unpasteurized milk from infected animals or, rarely, through laboratory exposure (Pustijanac et al. 2023; Martello et al. 2022). TBEV transmission is prevalent across endemic regions of Europe and Asia, especially in Central, Eastern, and Northern Europe, as well as parts of Siberia, the Russian Far East, and northern China, corresponding to the distribution of *I. ricinus* and *I. persulcatus* ticks.

TBEV invades the CNS through multiple mechanisms, primarily via the hematogenous route, while eliciting profound neuroinflammatory and immunomodulatory responses. Following CNS entry, TBEV induces a robust neuroinflammatory response characterized by the upregulation of cytokines and chemokines such as IL-6, IL-8, and RANTES (Pokorna Formanova et al. 2019; Zhang et al. 2016). These mediators drive leukocyte recruitment into the CNS, amplifying inflammation and potentially facilitating further viral entry via a Trojan horse mechanism, in which infected leukocytes cross the BBB and deliver virus into the CNS (Fig. 1C). Notably, activation of the interferon regulatory factor 3 (IRF-3) pathway enhances RANTES expression and contributes to the development of neuropathology.

TBEV also crosses the BBB by infecting BMECs, resulting in downregulation of tight junction proteins and increased paracellular permeability which facilitates basolateral release of infectious particles into the CNS without causing overt cytopathic effects (Mustafa et al. 2019). Infected endothelial cells, together with activated astrocytes and microglia, further compromise BBB integrity through the secretion of proinflammatory mediators, creating a permissive environment for viral entry and immune cell infiltration (Fig. 1A).

In parallel, TBEV displays strong neurotropism, directly infecting neurons and inducing apoptosis and cellular degeneration, hallmarks of TBEV-induced neurological disease. Neurons are particularly susceptible to TBEV-mediated cytopathic effects whereas astrocytes exhibit greater resistance to infection (Selinger et al. 2022). This cell-type-specific pathology is a key driver of disease severity and neurovirulence. TBEV infection modulates host gene expression through alterations in both mRNA and microRNA networks, affecting pathways involved in immune regulation and nervous system development (Selinger et al. 2022). By manipulating the host pre-mRNA splicing and miRNA profiles, TBEV dampens antiviral responses and enhance viral replication, factors that may underlie differences in strain virulence and disease severity.

##### Neurological manifestations of TBEV

The clinical manifestations of TBEV are diverse, ranging from mild, non-specific symptoms to severe neurological complications (Euringer et al. 2023; Beaute et al. 2018). The disease characteristically follows a biphasic pattern, initiating with febrile symptoms such as headache and myalgias, followed by significant neurological involvement characterized by conditions including meningitis, encephalitis, and myelitis (Euringer et al. 2023; Freimane et al. 2024). Advanced age, male sex, and pre-existing diabetes are risk factors for severe TBE (Radzisauskiene et al. 2020). In many instances, particularly among immunocompetent patients, neurological symptoms emerge following a symptom-free interval, during which viral neuroinvasion and immune-mediated injury may lead to severe outcomes such as focal neurological deficits or coma (Euringer et al. 2023; Beaute et al. 2018). These neurological patterns are influenced by the infecting TBEV subtype, with European strains more often following a biphasic course dominated by meningitis, Siberian strains tending toward monophasic illness with prolonged or relapsing neurological effects, and Far Eastern strains linked to aggressive encephalitis with frequent brainstem and spinal cord involvement, respiratory paralysis, and a substantially higher mortality risk (Chiffi et al. 2023). Encephalitis is the most common manifestation in adults, occurring in 61% of cases, while meningitis is more prevalent in children, affecting 77% of cases (Halsby et al. 2024). Radiculitis, characterized by nerve root inflammation, occurs in 14% of adult cases, while myelitis is less common, affecting 6% of adults and 1.3% of children (Halsby et al. 2024). Myelitis may present as poliomyelitis-like flaccid paralysis, sometimes involving respiratory muscles. Severe occipital headaches and seizures are also reported, with headaches being the most prevalent symptom in some studies (Fareed et al. 2024). Rare presentations include brainstem encephalitis with cranial nerve palsies, dysphagia, and respiratory compromise, as well as peripheral neuropathies without overt CNS involvement (Freimane et al. 2024; Chiffi et al. 2023). Infected cells exhibit both apoptotic and necrotic features, with ultrastructural changes such as chromatin condensation and cytoplasmic vacuolation (Ruzek et al. 2009). A significant proportion of TBEV cases require intensive care, with ICU admissions reported at 15.5% for adults and 16.4% for children (Halsby et al. 2024). While mortality is low at around 1%, long-term sequelae are common, affecting 40–50% of patients, often manifesting as post-encephalitic syndrome with headaches, fatigue, cognitive impairment, mood disorders, tremor, and ataxia, thereby significantly impairing quality of life (Chiffi et al. 2023; Halsby et al. 2024).

**Case studies/outbreaks of TBEV**

**Table:** 

Year	Location	Reported cases	Neuroinvasive cases	Deaths	Notable features	References
1951	Rožňava, Slovakia	~ 660	Not specified (CNS involvement suspected)	2	First and largest documents alimentary-associated outbreak caused by unpasteurized milk	Ruzek and Kaucka 2024
1969–1972	Prague, Czechoslovakia	633	All viral meningoencephalitis	5	12.8% experienced paresis	Duniewicz et al. 1975
1997–2008	Czech Republic	7288	None reported	0	64 foodborne cases associated with mostly milk/cheese	Kriz et al. 2009
1980–2021	Slovakia	43	12	0	Majority of neuroinvasive cases presented with meningoencephalitis	Elbaz et al. 2022
2012–2016	Europe	12,500	Not specified	< 1%	TBE became notifiable across EU; wide geographic reporting; Czech Republic and Lithuania accounted for ~ 39% of cases	Beauté et al. 2018
2016	Slovakia	44	Not specified	Not specified	~ 500 exposed; linked to sheep cheese consumption	Dorko et al. 2018
2016	Alsace and Alpine, France	54	46	0	Meningoencephalitis with altered consciousness	Velay et al. 2018

#### Dengue virus

##### Mechanisms of DENV CNS invasion

Dengue virus is primarily transmitted to humans through the bite of infected female *Aedes* mosquitoes, principally *Ae. aegypti* and, to a lesser extent, *Ae. albopictus* (Pourzangiabadi et al. 2025). Secondary transmission can also occur via blood transfusion, organ transplantation, and vertical transmission from mother to child, though these routes are less common (Gimenez-Richarte et al. 2022; Jayant et al. 2022; Lubis et al. 2025). Dengue is endemic in over one hundred countries across tropical and subtropical regions, with the highest burden in Southeast Asia, the Western Pacific, the Americas, and parts of Africa. While historically considered non-neurotropic, mounting evidence suggests that DENV crosses protective CNS barriers via multiple, interrelated mechanisms (Calderon-Pelaez et al. 2019). DENV can utilize both direct and indirect pathways for CNS invasion, including transcellular and paracellular routes, as well as the Trojan horse mechanism, where infected immune cells transport the virus across the BBB.

DENV infection of endothelial cells triggers proinflammatory cytokines (TNF-α, IL-6, IFN-γ) and chemokines (CXCL10, MCP-1), upregulates adhesion molecules (ICAM-1, VCAM-1), and promotes leukocyte recruitment and transmigration across the BBB (Puccioni-Sohler and Rosadas 2015). The Trojan horse mechanism involves the use of immune cells monocytes and macrophages, which are permissible to DENV, crossing the BBB and delivering the virus directly into the CNS (Fig. 1C) (Al-Obaidi et al. 2018). This method of invasion is particularly concerning as it allows DENV to evade the immune system and establish infection within the brain.

Upon gaining access to the CNS, in vitro studies demonstrate that DENV serotypes can induce disassembly of tight junction proteins claudin-1, occludin, ZO-1, and VE-cadherin, all of which are critical to maintaining BBB integrity (Fig. 1A) (Idris et al. 2019; Velandia-Romero et al. 2016). This disruption facilitates paracellular viral transit and permits immune cell infiltration, compounding neuroinflammation. Another proposed mechanism involves direct endothelial infection, whereby DENV replicates within human BMECs without necessarily inducing cytopathic effects. This suggests that the virus may traverse the BBB through a transcellular pathway, with progeny virions subsequently released basolaterally to infect glial cells and neurons (Mustafa et al. 2019; Velandia-Romero et al. 2016).

DENV NS1 protein, a secreted viral glycoprotein, further contributes to endothelial dysfunction. NS1 disrupts the endothelial glycocalyx layer, promotes vascular leak, and activates complement pathways, all of which lead to BBB structure and function alterations, further increasing susceptibility to CNS invasion and exacerbate neurological manifestations (Thomas 2015). Emerging evidence also suggests that BCSFB disruption may play a role in DENV invasion of the CNS (Thomas 2015). DENV antigen and RNA have been detected in choroid plexus epithelial cells and CSF in patients and experimental models, implying that this barrier may serve as another viral entry point into the CNS (Puccioni-Sohler and Rosadas 2015).

##### Neurological manifestations of DENV

DENV neurological involvement are estimated to occur in 0.5–7% of infected individuals (Carod-Artal 2014; Fong et al. 2024). Dengue encephalitis is one of the most reported presentations and is associated with altered consciousness, seizures, and focal neurological deficits (Harshani et al. 2024). Imaging reveals bilateral thalamic lesions, sometimes accompanied by hemorrhage or edema, while CSF analysis often shows mild lymphocytic pleocytosis and elevated protein levels (Lnu et al. 2022). Among children with serologically confirmed dengue, reported meningitis rates range from approximately 24% to 30%, whereas such cases are uncommon in adult (Soares et al. 2010). Meningitis due to DENV is less frequently reported but may occur either in isolation or in conjunction with encephalitis. In some cases, myelitis has been documented, including transverse myelitis with acute onset of lower limb weakness, sphincter disturbances, and sensory level abnormalities (Landais et al. 2019). MRI findings typically reveal hyperintense signals suggestive of inflammation in the spinal cord, particularly in the cervical and thoracic regions. Dengue-associated encephalopathy is often secondary to systemic illness and occurs more frequently than encephalitis in hospitalized patients with severe dengue (Trivedi and Chakravarty 2022). This condition is typically associated with hepatic dysfunction, metabolic disturbances (such as hyponatremia or hypoglycemia), cerebral edema, or multi-organ failure. It presents with altered mental status, confusion, and occasionally seizures, but without direct evidence of CNS viral replication (Patel et al. 2024). Stroke-like presentations, both ischemic and hemorrhagic, have also been reported which are largely attributed to thrombocytopenia, coagulopathy, and vasculitis associated with severe dengue, and they can lead to focal deficits and intracranial bleeding (Basnayake et al. 2021). Hypokalemic paralysis, a transient motor weakness resulting from sudden shifts in potassium levels, has been observed in some patients which typically resolves with potassium replacement and supportive care (Patel et al. 2024). In the post-acute phase, DENV has been implicated in several immune-mediated neurological disorders, with GBS being one of the most frequently reported, often appearing days to weeks after febrile illness (Fong et al. 2024). Patients present with ascending paralysis, areflexia, and albuminocytologic dissociation in the CSF. Electrophysiological studies typically show demyelinating features, though axonal variants have also been documented. Optic neuritis, retinal vasculopathy, and maculopathy have also been reported, often following systemic recovery (Carod-Artal 2014). Patients may present with visual field defects or even sudden vision loss which are believed to arise from immune-mediated inflammation or vascular leakage. Other immune-mediated complications include acute disseminated encephalomyelitis (ADEM) and mononeuropathies, such as cranial nerve palsies (Trivedi and Chakravarty 2022). These presentations suggest a post-viral autoimmune response against CNS myelin or neurons, possibly via molecular mimicry or dysregulated T-cell activation.

**Case studies/outbreaks of DENV**

**Table:** 

Year(s)	Location	Reported cases	Neuroinvasive cases	Deaths	Notable features	References
2001	India	3306	Not reported separately	53	Several dengue encephalitis and encephalopathy cases	NCVBDC n.d.; Kabilan et al. 2005
2005–2008	Brazil	209 suspected	8	4	High CNS involvement rate with encephalitis, meningitis reported	Araújo et al. 2012
2009	Brazil	1	1	0	Dengue meningitis without systemic dengue symptoms	Soares et al. 2010
2011	Sri Lanka	28,140	Not separately specified	185	Neurological complications during dengue epidemic	Gooneratne et al. 2014; Reller et al. 2012
2012	Central Brazil	498	28	2	Paresthesia, encephalitis, encephalopathy, seizure, meningoencephalitis, and paresis. DENV-3 was the predominant circulating serotype	Tassara et al. 2017
2012	Fortaleza, Ceará, Brazil	150	41	0	Encephalitis, meningoencephalitis, and meningitis	Araujo et al. 2012
2012	Jamaica	134	Not separately specified	5	Delayed presentation and short stature were associated with severe dengue, while sickle cell disease patients had longer hospital stays	Davidson and Christie 2017
2013	Singapore	22,170	0.5–21%	5	Dengue encephalitis, encephalopathy, neuromuscular issues. Mortality more in patients older than 60 years	Hapuarachchi et al. 2016; Shi et al. 2016; Carod-Artal et al. 2013;
2014–2015	Minas Gerais, Brazil	34,996 22	5.6% 5	Not specified -	Adults aged 20–39 were most affected, with higher incidence in women Pediatric CNS involvement with DENV-1, −2, −3 and co-infections	Tassara et al. 2017; da Silva et al. 2021; Marinho et al. 2017
2015	Malaysia	~ 120,000 116	Not specified 92	336	Encephalitis, transverse myelitis Encephalopathy, encephalitis, transverse myelitis, and dengue associated muscle dysfunction	Chem et al. 2024; Suppiah et al. 2018; Misra et al. 2015
2016	Odisha, India	8,304	12 encephalopathy cases	1	Encephalopathy and seizures during dengue outbreak	Swain et al. 2019; Bhuyan et al. 2025
2019	Philippines	> 430,000	Not specified	1612	Encephalopathy, encephalitis, seizures, GBS, ADEM, myelitis, and neuromuscular complications like myositis	Ong et al. 2022; Government of the Philippines 2020

#### Yellow fever virus

##### Mechanisms of YFV CNS invasion

Yellow fever virus is transmitted to humans primarily through the bite of infected *Aedes* and *Haemagogus* mosquitoes, with transmission occurring within sylvatic, intermediate, and urban cycles (Kuno 2024). *Ae. aegypti* is the principal vector for urban transmission, while *Haemagogus* spp. and *Ae. africanus* maintain sylvatic transmission in South America and Africa, respectively. Yellow fever is endemic in tropical regions of Africa and South America, with major burdens in countries such as Angola, Democratic Republic of Congo, Nigeria, Ghana, Brazil, and Peru, whereas it has been considered eradicated in the continental United States (Srivastava et al. 2024).

Although Yellow fever virus primarily targets hepatic and systemic tissues, some reports of neurological complications indicate that the virus can access the CNS (de Rezende et al. 2022). The well-characterized route is hematogenous dissemination, in which virus circulating at high titers crosses the BBB. YFV can infect BMECs, leading to barrier disruption or basolateral release of viral particles (Fig. 1A) (Mustafa et al. 2019). Viral entry also occurs via paracellular routes, where tight junctions between endothelial cells are disrupted by viral proteins such as NS1 or by host inflammatory cytokines including TNF-α and IL-6, creating openings that permit viral passage (de Sousa et al. 2024).

Another mechanism is the “Trojan horse” pathway (Fig. 1C), in which infected leukocytes migrate across the BBB, carrying virus into the CNS (Marshall et al. 2022). Infection of BMECs also activates astrocytes and microglia, which release inflammatory mediators that recruit additional immune cells to the CNS (Mustafa et al. 2019). This immune cell infiltration then exacerbates BBB breakdown and facilitates further viral entry, contributing to neuropathology.

Transneural or axonal spread also provides an alternative pathway for CNS invasion (Fig. 1D), allowing YFV to reach neural tissues by traveling along peripheral nerves and bypassing the BBB entirely (De Vries and Harding 2023). Once inside the CNS, YFV induces neuropathogenesis through multiple mechanisms, including direct cell lysis, cell cycle arrest, apoptosis, and immune-mediated damage.

##### Neurological manifestations of YFV

YFV-induced neurological manifestations are relatively uncommon but can present with a broad clinical spectrum. Reported symptoms range from mild, nonspecific presentations such as headache, dizziness, and confusion to severe, life-threatening conditions including encephalitis, meningoencephalitis, seizures, and coma (McGuinness et al. 2017; Beirao et al. 2017; Almeida Bentes et al. 2019). Neurological involvement may occur during the acute viremic phase or as a delayed complication, sometimes associated with immune-mediated processes. Children with YFV detected in cerebrospinal fluid have a 53% higher risk of developing neurological complications (Bentes et al. 2021). Autopsy studies have shown that YFV can cause perivascular inflammatory infiltrate in the brain, which is a significant neuropathological finding associated with the virus (Frassetto and Rosemberg 2023). This inflammation is likely due to immune activation and vascular changes induced by the virus. In severe neuroinvasive disease, patients may exhibit altered mental status, focal neurological deficits, ataxia, and signs of increased intracranial pressure (de Rezende et al. 2022; Beirao et al. 2017). Although highly effective, yellow fever vaccination has on rare occasions been associated with two severe adverse events: yellow fever vaccine–associated neurotropic disease (YEL-AND) and yellow fever vaccine–associated viscerotropic disease (YEL-AVD) (Martins et al. 2014). YEL-AND typically occurs within 4–30 days post-vaccination and may present as meningoencephalitis, GBS, or ADEM, particularly in primary vaccine recipients (Bayao et al. 2018). YEL-AVD, although even rarer, involves systemic viral dissemination resembling severe wild-type YFV and carries a high fatality rate. YEL-AVD can also lead to multiorgan failure and death, as observed in a 22-year-old female patient with high-titer vaccine viremia (Belsher et al. 2007). Seizures have been reported in both adult and pediatric cases, often correlating with neuroimaging or CSF findings consistent with encephalitis (Gerin et al. 2014). Histopathological studies in fatal YEL-AND and YEL-AVD cases have revealed neuronal degeneration, microglial activation, perivascular inflammatory infiltrates, and in some cases, viral antigen detection in brain tissue (Frassetto and Rosemberg 2023).

Regarding long-term neurological sequelae, follow-up data from YFV outbreak settings are scarce, making it difficult to determine the true prevalence or characteristics of persistent deficits. In a 10-year French surveillance study, the combined incidence of YEL-AND and YEL-AVD was estimated at 0.6 cases per 100,000 doses administered (Le Hir et al. 2024). YEL-AND cases generally had favorable clinical outcomes, whereas YEL-AVD cases were often life-threatening or fatal. Notably, some affected individuals harbored neutralizing autoantibodies against type I interferons, suggesting a potential defect in innate antiviral immunity.

**Case studies/outbreaks of YFV**

**Table:** 

Year(s)	Location	Reported cases	Neuroinvasive cases	Deaths	Notable features	References
1969	Nigeria	> 100,000	Not separately specified	40%	CNS signs in 25% of patients, including generalized seizures	Carey et al. 1972
2001–2002	USA	6	4	0	YEL-AVD and YEL-AND, all recovered without sequelae	CDC 2002 (Prevention 2002)
2007	USA	15	9	0	Headache and encephalitis presentations	McMahon et al. 2007
2012	Darfur, Sudan	844	Not specified	171	Highest fatality rate was observed in children under two years old	Markoff 2013; Alhakimi et al. 2015
2012–2014	South Omo, Ethiopia	165	Not specified	62	Majority of cases in rural areas; high mortality among males aged 15–44	Mulchandani et al. 2019
2017	São Paulo, Brazil	1	1	1	Encephalitis	Marinho et al. 2019
2007–2022	France	67	67	-	All YEL-AND cases. Highest rate in children 5–9 years	Martins et al. 2014

#### Powassan Virus

##### Mechanisms of POWV CNS invasion

Powassan virus is transmitted to humans primarily through the bite of infected *Ixodes* ticks, most notably *I. scapularis* and *I. cookei*, with transmission possible within minutes of attachment (Obellianne et al. 2024). These ticks maintain the virus in enzootic cycles involving small mammals such as rodents and groundhogs. POWV is endemic in parts of Russia and North America, including the northeastern United States, the Great Lakes region, and eastern Canada (Kapoor and Zash 2025). Although diagnosed/confirmed human cases are rare, infection is often associated with severe neurologic disease, long-term sequelae, and a mortality rate of approximately 10% (Goethert et al. 2021).

POWV neuroinvasion is a multifactorial process influenced by viral dose, lineage, and host factors. The two recognized POWV lineages of lineage I (classic Powassan virus) and lineage II (deer tick virus) exhibit distinct patterns of CNS entry and tropism resulting in distinct clinical manifestations and immune responses (Reynolds et al. 2024). Experimental evidence demonstrates that the efficiency and route of neuroinvasion are dose-dependent (Santos et al. 2021). Low-dose infections typically require the presence of tick salivary gland components to establish CNS infection, whereas high-dose infections can achieve CNS spread independently of salivary factors, following a caudal-to-rostral pattern in the brain (Santos et al. 2021).

At the cellular level, POWV is also capable of cell-to-cell transmission and can establish persistent infection in human brain microvascular endothelial cells and pericytes (Conde et al. 2022). These findings suggest that the virus may cross the BBB via nonlytic basolateral spread, moving directly from infected endothelial cells to adjacent pericytes without causing immediate cytopathic effects.

##### Neurological manifestations of POWV

Clinical presentations of POWV range from asymptomatic infections to fatal encephalitis, with neuroinvasive disease showing high morbidity and mortality rates (Ndukwe et al. 2024; Mendoza et al. 2024). The most common neurological signs and symptoms include severe occipital headache, seizures, and flaccid paresis (Fareed et al. 2024). Clinical syndromes encompass rhombencephalitis, isolated meningitis, meningoencephalitis, meningoencephalomyelitis, and opsoclonus myoclonus syndrome (Mendoza et al. 2024). Mortality occurs in approximately nineteen percent of neuroinvasive cases, with seventy-three percent of survivors experiencing residual neurological deficits (Mendoza et al. 2024). Mouse models demonstrate POWV's strong neuronal tropism, causing meningoencephalitis with perivascular infiltration and a poliomyelitis-like syndrome affecting spinal cord ventral horns (Santos et al. 2016). POWV causes long-term neurological sequelae in approximately half of confirmed cases and death in over ten percent (Campbell and Krause 2020).

**Case studies/outbreaks of POWV**

**Table:** 

Year(s)	Location(s)	Reported cases	Neuroinvasive cases	Deaths	Notable features	References
1999–2005	USA	9	8	0	Profound muscle weakness, confusion, encephalitis	Hinten et al. 2008
2006–2016	USA	99	89	11	Encephalitis, meningitis and febrile illness. 90% cases hospitalized	Krow-Lucal et al. 2018
2010–2020	Minnesota, Wisconsin, and New York, USA	194	Not specified	22	Encephalitis reported	Krow-Lucal et al. 2018
2020	Northern Wisconsin, USA	1	1	1	Fever, acute mental status change, slurred speech, falls, and left arm weakness	Yu et al. 2020
2024	USA	1	1	1	Fever, myalgia, and confusion and seizures	Carrasco and Horiszny 2024

#### Saint Louis encephalitis virus (SLEV)

##### Mechanisms of SLEV CNS invasion

Saint Louis encephalitis virus is transmitted to humans primarily through the bite of infected *Culex* mosquitoes, notably *C. pipiens*, *C. quinquefasciatus*, and *C. tarsalis* (Danforth et al. 2022). These mosquitoes maintain the virus in enzootic cycles involving birds as amplifying hosts, while humans are incidental hosts (Danforth et al. 2022). SLEV is endemic throughout much of the Americas, particularly in the United States, with cases concentrated in the Mississippi River basin, the Gulf Coast, and parts of the Midwest (Diaz et al. 2018).

SLEV employs multiple, complementary strategies to gain access to the CNS, reflecting its complex neuropathogenesis. One primary route of CNS entry is via the olfactory pathway (Fig. 1F). After peripheral exposure, the virus infects olfactory neuroepithelial cells in the nasal cavity, which project directly into the olfactory bulb (Monath et al. 1983). Through retrograde axonal transport, SLEV travels along these neurons, establishing infection within the CNS even in the context of low systemic viremia. This pathway effectively bypasses the BBB and may account for the early onset of neurological symptoms observed in infected individuals.

In addition to olfactory invasion, SLEV can penetrate the CNS through hematogenous spread by compromising the integrity of the BBB. The virus infects endothelial cells and disrupts tight junctions, facilitating the passage of viral particles and inflammatory mediators into the CNS (Fig. 1A) (Vries And Harding 2023). This mechanism contributes to widespread neural infection and amplifies neuroinflammation, exacerbating neurological dysfunction.

SLEV also exploits axonal transport along peripheral nerves as an alternative CNS entry route. By traveling retrogradely along sensory or motor neurons, the virus can reach the spinal cord or brain without overtly disrupting the BBB, providing an explanation for focal neurological deficits such as paralysis observed in rare cases (Vries And Harding 2023). Once within the CNS, SLEV directly infects neurons and glia, leading to inflammation, excitotoxicity, and neural apoptosis.

##### Neurological manifestations of SLEV

SLEV infection presents a spectrum of neurological manifestations, ranging from meningitis to severe encephalitis. Acute encephalitis typically presents with fever, headache, confusion, and tremors, often preceded by a flu-like prodrome (Simon And Graham 2021). Meningitis may be characterized by stiff neck, disorientation, photophobia, confusion, and language impairment, with MRI and EEG studies revealing temporal lobe involvement (Seijo et al. 2011). Rarely, focal neurological deficits such as paralysis and delirium have been reported, reflecting localized brain involvement (Naidech and Elliott 1999). Post-infectious encephalomyelitis can also develop following mild SLEV infection, manifesting as weakness and altered mental status, suggesting that immune-mediated neurological complications may arise after the acute phase (Sejvar et al. 2004). SLEV is not restricted to humans and has been isolated from a horse with neurological signs in Brazil (Rosa et al. 2013). Experimental studies in mice have further explained SLEV neuropathogenesis, demonstrating neurodegeneration in the motor cortex, corpus striatum, cerebellum, and other brain regions, accompanied by apoptosis and excitotoxic-like neuronal damage (Rivarola et al. 2018).

**Case studies/outbreaks of SLEV**

**Table:** 

Year(s)	Location(s)	Reported cases	Neuroinvasive cases	Deaths	Notable features	References
1932	Illinois, USA	38	Not separately specified	14	Fever, headache, confusion, and seizures; severe encephalitis in fatal	Diaz et al. 2018
1933	St Louis, Missouri, USA	1097	Not separately specified	201	Encephalitis, meningitis, headache, confusion, photophobia; high case-fatality in children and elderly	Chamberlain 1980
1975	Mississippi, USA	229	133	36	Encephalitis and aseptic meningitis	Powell and Blakey 1977
1990–1991	Florida, USA	226	Not separately specified	11	Encephalitis, meningitis, febrile illness; headache, confusion, photophobia	Day and Stark 2000; Day 2001
2001	Louisiana, USA	63	63	3	Fever, meningitis, altered mental status, tremors	Jones et al. 2002
2005	Argentina	47	Not separately specified	9	Headache, sensory depression, temporal–spatial disorientation, tremors, and changes in consciousness,	Spinsanti et al. 2008
2010	Buenos Aires, Argentina	13	7	0	Meningitis and encephalitis	Seijo et al. 2011
2003–2017	Arizona, USA	193	148	11	Encephalitis, meningitis and febrile illness	Curren et al. 2018

### Family Togaviridae

#### Eastern Equine encephalitis virus (EEEV)

##### Mechanisms of EEEV CNS invasion

Eastern equine encephalitis virus is transmitted to humans primarily through the bite of infected mosquitoes, with the virus maintained in an enzootic cycle between birds and *Culiseta melanura* (Armstrong and Andreadis 2022). Spillover to humans occurs via bridge vectors such as *Aedes*, *Coquillettidia*, and *Culex* species, which feed on both birds and mammals (Corrin et al. 2021). EEEV is endemic to eastern North America, particularly the Atlantic and Gulf Coast states and the Great Lakes region (Corrin et al. 2021).

The mechanisms EEEV exploits involve specific mechanisms to cross the BBB and disseminate within the CNS, leading to severe neurological outcomes. One mechanism of EEEV neuroinvasion is a vascular route of CNS entry, with initial replication in peripheral tissues, particularly osteoblasts, leading to high-titer viremia (Vogel et al. 2005). The virus employs caveolin-1 (Cav-1)-mediated transcytosis, a vesicular transport process that shuttles viral particles across brain endothelial cells (Fig. 1B). This entry mechanism is tightly regulated by Rho GTPases and can be curtailed by type I interferons, which restrict viral replication in endothelial cells, pericytes, and astrocytes, highlighting the interplay between viral strategies and host defenses (Salimi et al. 2020).

EEEV is a highly pathogenic virus classified as a select agent with potential biothreat use due to its ability to cause severe disease following aerosol exposure (Phelps et al. 2019). Following aerosol exposure, EEEV infects olfactory sensory neurons within the nasal epithelium (Fig. 1F), a specialized neuronal population whose dendrites are directly exposed to the external environment (Honnold et al. 2015; Williams et al. 2022). These neurons project unmyelinated axons through the cribriform plate to the olfactory bulb, providing a direct anatomical route to the CNS that circumvents the BBB. Upon entry into olfactory neurons, EEEV exploits host intracellular trafficking machinery to undergo retrograde axonal transport along microtubules, likely mediated by dynein-dependent motor complexes, enabling efficient movement of viral particles from peripheral neuronal termini to neuronal cell bodies within the olfactory bulb. From this primary site of infection, EEEV rapidly spreads transsynaptically and along anatomically connected neuronal circuits to deeper brain regions (Fig. 1D), facilitating widespread CNS dissemination early in infection (Honnold et al. 2015; Williams et al. 2022).

##### Neurological manifestations of EEEV

Clinical presentations of EEEV neuroinvasive disease range from mild encephalopathy to coma, with common symptoms including altered mental status, seizures, and focal neurological deficits (Garcia-Dominguez et al. 2024). The onset is often abrupt, beginning with a short prodromal phase of fever, malaise, and headache, followed by rapid progression to encephalitis characterized by high fever, confusion, and meningeal irritation (Lindsey et al. 2018). Seizures are frequent, often severe or refractory, reflecting the virus’s pronounced neuronal tropism and its ability to disrupt cortical function. Neuropathological findings demonstrate significant inflammation and neuronal injury, including lymphocytic infiltrates, gliosis, and widespread neuronal necrosis (Williams et al. 2022; Albe et al. 2021). Viral replication occurs predominantly in neurons of the cerebral cortex, hippocampus, thalamus, and brainstem, leading to edema and perivascular inflammation (Williams et al. 2022). This neuronal targeting underlies the cognitive impairment, movement disorders, and behavioral changes often seen in survivors. In children, the disease tends to manifest more aggressively, with fulminant seizures and a higher incidence of long-term deficits, including intellectual disability, motor dysfunction, and personality changes (Lindsey et al. 2018; Ronca et al. 2016). The case fatality rate for neuroinvasive EEEV is approximately 30%, and over 50% of survivors experience chronic neurological sequelae affecting cognitive, sensory, and motor functions (Lindsey et al. 2018). Diagnosis typically relies on CSF analysis and neuroimaging, with MRI findings frequently showing basal ganglia and thalamic involvement (Garcia-Dominguez et al. 2024). Long-term neurological sequelae can persist after acute infection, affecting cognitive, sensory, and motor functions (Ronca et al. 2016).

**Case studies/outbreaks of EEEV**

**Table:** 

Year(s)	Location	Reported cases	Neuroinvasive cases	Deaths	Notable features	References
1938	Massachusetts, Connecticut, Rhode Island, USA	38	Not specified	25	First documented human outbreak; simultaneous equine and pheasant epizootics	Langsjoen et al. 2023
1988–1994	Jamaica, Dominican Republic	36	36	Not specified	Neurological manifestations included confusion, somnolence, focal weakness, seizures, and meningeal signs	Sah et al. 2023
2010	Panama	13	7	3	19 hospitalized patients with encephalitis, 7 had confirmed EEE, 3 had VEE, and 1 was infected with both viruses	Carrera et al. 2013
2019	Massachusetts, Michigan, Connecticut, New Jersey, Rhode Island, North Carolina, Tennessee, USA	34	34	12	Largest multistate outbreak in decades; median age 64; case-fatality 35%; higher mortality ≥ 70 yrs	Lindsey et al. 2020; Brown et al. 2021; Hill et al. 2023
2019	Alabama, USA	1	1	1	Fatal case; genomic analysis showed serum vs. CSF viral variation	Hughes et al. 2021
2019	New Jersey, USA	1	1	0	42-year-old male; severe encephalitis; prolonged ICU stay; recovery with rehab	Millet et al. 2021
2024	New Hampshire, USA	1	1	0	Severe case; father critically ill with EEEV and coinfections	Keane 2024
2024	Connecticut, USA	1	1	1	Backyard exposure; first EEEV death in CT in a decade	Donlevy 2024

#### Western Equine encephalitis virus (WEEV)

##### Mechanisms of WEEV CNS invasion

Western equine encephalitis virus is maintained in enzootic cycles between passerine birds and *Culex tarsalis* mosquitoes, with humans and horses serving as dead-end hosts during occasional spillover events, although disease severity is generally lower than EEEV (Lindsey et al. 2018; Ciota 2022). *Aedes* species as bridge vectors, transmitting the virus to humans and horses. WEEV is endemic to North America, historically most prevalent in the western and central United States, particularly the Mississippi Valley and western plains (Lindsey et al. 2018; Ciota 2022).

WEEV has been shown to exploit caveola-mediated transcytosis (Cav-MT) to traverse an intact BBB (Salimi et al. 2020). This process, dependent on caveolin-1 in brain microvascular endothelial cells, is negatively regulated by type I interferon signaling and Rho kinase inhibitors. Importantly, viral passage via Cav-MT occurs without active replication in endothelial cells, suggesting a non-cytopathic but effective entry strategy in immunocompetent hosts (Phillips et al. 2016).

Beyond vascular routes, WEEV spreads via neuronal routes similar to EEEV (Fig. 1A, D & F). WEEV infect olfactory and trigeminal sensory neurons, enabling direct spread from the olfactory bulb to other brain regions, such as the entorhinal cortex and hippocampus (Phillips et al. 2013). Bioluminescence imaging has shown that WEEV enters the CNS through circumventricular organs (CVOs) such as the area postrema, median eminence, pineal body, where the BBB is naturally absent, followed by spread along the neuronal axis (Phillips et al. 2016). Once in the CNS, WEEV provokes a strong neuroinflammatory response characterized by rapid induction of cytokines and chemokines, activation of glial cells, and recruitment of peripheral immune cells (Bantle et al. 2019). This inflammatory milieu not only disrupts BBB integrity but also drives neuropathology, including selective loss of dopaminergic neurons and abnormal protein aggregation, ultimately contributing to long-term neurological impairments (Bantle et al. 2019).

##### Neurological manifestations of WEEV

WEEV infection exhibits a spectrum of neurological outcomes ranging from subclinical involvement to severe encephalitis. The severity of disease is strongly influenced by age and immune status, with children, the elderly, and immunocompromised individuals being most susceptible to CNS disease (Simon et al. 2025). The hallmark clinical presentation is acute encephalitis, presenting symptoms such as confusion, somnolence, and coma, particularly in vulnerable populations like infants and older adults. Seizures are common, particularly in pediatric cases, and may occur early in the disease course (Simon et al. 2025; Saul et al. 2025). In some patients, infection manifests as meningoencephalitis, with additional signs of meningeal irritation such as neck stiffness and photophobia. Rarely, involvement of deep brain structures such as the basal ganglia and substantia nigra has been associated with movement disorders, including tremor and parkinsonian features (Bantle et al. 2019).

Neuropathological studies from fatal human cases and experimental models provide insight into the basis of these clinical features. Typical findings include perivascular cuffing, neuronal necrosis, gliosis, and microglial activation in the cerebral cortex, hippocampus, thalamus, and brainstem (Phillips et al. 2016). Severe cases may also show hemorrhagic lesions (Simon et al. 2025). Notably, selective involvement of the substantia nigra in murine models has been proposed as a mechanistic explanation for post-encephalitic movement abnormalities (Salimi et al. 2020). Survivors of WEEV encephalitis often experience persistent neurological impairments (Bantle et al. 2019). Reported sequelae include cognitive deficits, learning disabilities, behavioral changes, motor dysfunction, and epilepsy (Ronca et al. 2016). Longitudinal studies suggest that 30–50% of pediatric survivors exhibit permanent neurological or developmental complications (Simon et al. 2025). These long-term outcomes highlight the substantial public health burden associated with even rare outbreaks. Animal models mirror many of the clinical manifestations observed in humans. Experimental WEEV infection in mice produces seizures, tremors, and ataxia, correlating with viral replication in hippocampal and cortical neurons (Phillips et al. 2016). WEEV infection also causes selective loss of dopaminergic neurons, neuroinflammation, and widespread protein aggregation, paralleling parkinsonian features documented in human cases (Bantle et al. 2019). Mechanistically, neurological disease reflects a combination of direct viral cytotoxicity and immune-mediated injury, as proinflammatory cytokines such as TNF-α, IL-6, and chemokines are elevated in infected brains (2020).

**Case studies/outbreaks of WEEV**

**Table:** 

Year(s)	Location(s)	Reported cases	Neuroinvasive cases	Deaths	Notable features	References
1941	Canada	~ 3400	Not specified	Not specified	Early documented human cases; equine epizootic led to virus identification	CDC1987 (Prevention 1981)
1977	USA	41	Not specified	Not specified	Largest outbreak prior to 1941	CDC1987 (Prevention 1981)
1975–1978	USA	947	Not specified	Not specified	Rare sporadic cases; improved surveillance identified subclinical infections	Monath 1979
1981	Colorado, Nebraska, Texas, North Dakota, and Montana, USA	37	Not specified	1	Cases mostly in children; neuroinvasive manifestations included seizures and tremors	CDC1987 (Prevention 1981)
2009	Uruguay	1	1	1	Fatal human case; first reported human WEEV case in Uruguay in over a decade; rural exposure	Delfraro et al. 2011
2023–2024	Argentina, and Uruguay	217	Not specified	12	Outbreaks in humans and equines; vector control and surveillance efforts ongoing	Campos et al. 2024

#### Venezuelan equine encephalitis virus (VEEV)

##### Mechanisms of VEEV CNS invasion

Venezuelan equine encephalitis virus exhibits enzootic circulation primarily between rodents and *Culex (Melanoconion)* spp. mosquitoes, whereas during epizootic outbreaks, *Aedes* and other mosquito species act as bridge vectors, transmitting the virus to horses (amplifying hosts) and humans (dead-end hosts) (Hoyer et al. 2019; Deardorff et al. 2011). VEEV is endemic in Central and South America, with outbreaks reported in Venezuela, Colombia, Ecuador, Peru, and Trinidad, often occurring in cycles every 5–10 years (Guzman-Teran et al. 2020).

Following mosquito transmission, VEEV undergoes initial replication in dermal dendritic cells and lymphoid tissues, producing a high-titer viremia that facilitates systemic dissemination (Sharma and Knollmann-Ritschel 2019). The virus primarily enters the brain through hematogenous spread by infecting and disrupting the brain microvascular endothelial cells, leading to increased permeability of the BBB (Sharma and Knollmann-Ritschel 2019). Inflammatory mediators such as TNF-α, IL-6, and MMPs further weaken barrier integrity, allowing viral entry along with leukocyte infiltration (Phillips et al. 2016). Recent evidence suggests that VEEV, like WEEV, can cross an otherwise intact BBB through caveola-mediated transcytosis (Cav-MT). This process depends on caveolin-1 activity in endothelial cells and occurs without active viral replication within these cells (Salimi et al. 2020; Phillips et al. 2016). Type I interferon signaling restricts endothelial infection, reinforcing the likelihood that Cav-MT serves as a non-cytolytic but effective entry route (Salimi et al. 2020). CVOs have been identified as early targets of VEEV entry, with reporter virus imaging in mice highlighting infection of the area postrema, pineal gland, and median eminence, followed by spread into adjacent neuronal networks (Phillips et al. 2016). VEEV also exploits neuronal pathways to gain CNS access (Salimi et al. 2020). Experimental models demonstrate invasion via the olfactory epithelium, with the virus traveling retrogradely along olfactory sensory neurons into the olfactory bulb (Durrant et al. 2016; Cain et al. 2023).

At later stages of infection, proinflammatory cytokines and chemokines significantly enhance BBB permeability, amplifying viral entry and immune cell infiltration (Schafer et al. 2011). Toll-like receptor 4 (TLR4) plays a critical role in mediating BBB permeability during VEEV infection, with TLR4-deficient mice showing reduced barrier compromise and lower expression of matrix metalloproteinases, chemokines, and inflammatory cytokines (Hollidge et al. 2021). This suggests that early invasion occurs through selective mechanisms such as Cav-MT and neuronal routes, while secondary waves of entry are driven by immune-mediated barrier. Elevated CNS cytokine levels, including TNF-α and IL-6, indicate that both direct viral cytotoxicity and immune-mediated inflammation contribute to neuropathogenesis of VEEV (Salimi et al. 2020).

##### Neurological manifestations of VEEV

VEEV infection can range from subclinical disease to severe neuroinvasive illness, particularly in children, the elderly, and immunocompromised individuals (Sharma and Knollmann-Ritschel 2019). VEEV’s pronounced neurotropism is reflected in the spectrum of acute neurological syndromes, neuropathological changes, and long-term sequelae. Acute encephalitis is the most frequent manifestation, presenting with fever, headache, lethargy, confusion, and in severe cases, stupor or coma (Ronca et al. 2016). Seizure activity is also common, especially in pediatric patients, and may precede or accompany other signs of CNS involvement (Ronca et al. 2016). Viral replication in the basal ganglia and brainstem can lead to tremors, ataxia, and other movement abnormalities, reflecting disruption of motor circuits leading to movement and motor disorders (Williams et al. 2023). The virus can cause lasting neurological deficits through severe neuroinflammatory responses that promote neuronal injury, including selective loss of dopaminergic neurons and widespread protein aggregation (Bantle et al. 2019). Some patients exhibit meningoencephalitis with signs of meningeal irritation, including neck stiffness and photophobia (Ronca et al. 2016). Observed lesions include neuronal necrosis in the cerebral cortex, hippocampus, thalamus, and brainstem; perivascular cuffing and leukocyte infiltration, reflecting inflammatory responses; gliosis and microglial activation, indicative of innate immune activation and tissue repair; and hemorrhagic lesions in severe or fatal cases (Phillips et al. 2016). Survivors of VEEV encephalitis may experience persistent neurological deficits, such as cognitive impairments and learning difficulties, motor deficits (tremor, ataxia, or fine motor dysfunction), seizure disorders or epilepsy, and behavioral and psychiatric disturbances (Ronca et al. 2016). Longitudinal studies suggest that a significant proportion of survivors, particularly children, display lasting neurocognitive and motor impairments, highlighting the long-term burden of VEEV outbreaks (Chow and Dehority 2021).

**Case studies/outbreaks of VEEV**

**Table:** 

Year(s)	Location(s)	Reported cases	Neuroinvasive cases	Deaths	Notable features	References
1962–1964	Venezuela & Colombia	> 200,000	960	56	Severe encephalitis, seizures, ataxia, tremors; significant pediatric involvement	Aguilar et al. 2011
1969–1972	Southern Mexico (Chiapas, Oaxaca, Campeche, Guerrero)	~ 52,000	93	93	Encephalitis, seizures, ataxia, confusion; high	Estrada-Franco et al. 2004
1995	Venezuela & Colombia	14,156	Data not specified	26	Acute febrile illness progressing to encephalitis in humans; high equine morbidity	Molina et al. 1999
2010	Panama	99	Data not specified	Data not specified	19 hospitalized; Severe neurological symptoms including meningoencephalitis in hospitalized patients	Carrera et al. 2020
2016–2017	Peru & Ecuador	~ 200	Data not specified	2	Neurological involvement included seizures, confusion, and tremors in severe cases	Vilcarromero et al. 2010

### Family Peribunyaviridae

#### La Crosse Virus (LACV)

##### Mechanisms of LACV CNS invasion

La Crosse virus, a member of the California serogroup of orthobunyaviruses is transmitted to humans primarily through the bite of infected *Aedes triseriatus* mosquitoes, with the virus maintained in an enzootic cycle involving small mammals such as chipmunks and squirrels (Goldman and Hamer 2024). Other *Aedes* species, including *Ae. albopictus* and *Ae. japonicus*, may also serve as vectors. LACV is endemic in the United States, especially in the Midwest, Mid-Atlantic, and Appalachian regions, and is the leading cause of pediatric arboviral encephalitis in the country (Matthews et al. 2022).

Although studies on LACV replication are limited, animal models indicate that the virus initially replicates in peripheral tissues, then enters the bloodstream, infects the nasal turbinates, and eventually reaches the central nervous system, likely via the olfactory neurons (Bennett et al. 2008; Wilson et al. 2021). LACV primarily invades the CNS via the olfactory bulb, where capillaries are highly susceptible to virus-induced vascular leak, permitting viral particles to enter the brain parenchyma (Winkler et al. 2015). Notably, the entry does not rely on axonal transport from olfactory sensory neurons but instead depends on compromised capillary integrity in specific brain regions (Winkler et al. 2015).

At the cellular level, LACV enters neurons through Clathrin-mediated endocytosis, requiring trafficking into early endosomes via Rab5-dependent pathways (Hollidge et al. 2012). The viral fusion peptide within the Gc glycoprotein is a critical determinant of neuroinvasion; mutations in this region attenuate the virus’s ability to invade the CNS peripherally, while neurovirulence remains intact when the virus is directly introduced into brain tissue (Hollidge et al. 2021). Hematogenous dissemination is also a key route for CNS entry. LACV infects brain microvascular endothelial cells directly and induces the release of inflammatory mediators such as TNF-α, IL-6, and MMPs, which compromise BBB integrity (Basu et al. 2021; Basu et al. 2023). Increased vascular permeability allows both viral particles and immune cells to access the CNS, potentially amplifying neuroinvasion.

##### Neurological manifestations of LACV

Children under 16 years are most commonly affected by neuroinvasive LACV, often presenting with more severe CNS involvement than adults, including status epilepticus, seizures, and cognitive deficits (Ouellette 2024). Clinical manifestations range from mild aseptic meningitis to severe encephalitis, with headache, fever, vomiting, lethargy, irritability, ataxia, focal motor deficits, and altered mental status frequently observed (Feng et al. 2025; Boutzoukas et al. 2023). Seizures, confusion, coma, paralysis and altered mental status at presentation are independent predictors of severe disease, and epileptiform discharges on electroencephalogram may predict long-term epilepsy (Ouellette 2024; Boutzoukas et al. 2023). Long-term neurological sequelae are common and may be under-recognized, including epilepsy, hemiparesis, cognitive and neurobehavioral abnormalities, and fine motor deficits, with severity correlating with the extent of acute CNS involvement (Boutzoukas et al. 2023). Although mortality is low in immunocompetent children, severe LACV encephalitis can result in life-threatening complications such as cerebral edema, and persistent neurological disability, highlighting the virus's neurotropic potential (Bennett et al. 2008; Boutzoukas et al. 2023).

**Case studies/outbreaks of LACV**

**Table:** 

Year(s)	Location(s)	Reported cases	Neuroinvasive cases	Deaths	Notable features	References
1964 −1995	USA	2245	Not specified	Not specified	Pediatric encephalitis, seizures, lethargy, ataxia reported	Foster et al. 2007
1980	Quebec, Canada	3	3	0	Clinical encephalitis, fever, and seizures	Fauvel et al. 1980
1981–1983	Ohio, USA	81	Not specified	Not specified	Seizures and coma in severe pediatric cases	Ohio Department of Health 2024
1987	West Virginia, USA	19	11	1	All patients < 15 years old; Meningitis and meningoencephalitis	CDC 1988
2004 −2024	USA	1503	1395	15	Encephalitis and meningitis	CDC 2025

#### California Encephalitis Virus (CEV)

##### Mechanisms of CEV CNS invasion

California encephalitis virus is transmitted to humans primarily through the bite of infected *Aedes* and *Ochlerotatus* mosquitoes, which maintain the virus in an enzootic cycle with small mammals such as rodents while humans are incidental hosts (Reeves and Hammon 1952; Reeves et al. 1983). CEV is endemic in North America, particularly in the Midwest, Great Lakes, and Appalachian regions of the United States, with most cases occurring in children (McJunkin et al. 1998; Reimann et al. 2008).

Mechanistic studies on CNS invasion are extremely limited for CEV itself. Most experimental work has focused on LACV, a member of the same California serogroup of Orthobunyaviruses. Inferences from studies on other encephalitis viruses suggest that CEV may reach the CNS through multiple complex mechanisms. One key route involves the alteration of tight junction proteins that maintain BBB integrity. CEV causes modifications in these proteins, resulting in increased permeability of the BBB resulting in virus entry into the neural tissues (Al-Obaidi et al. 2018). CEV also gains cellular entry through adsorptive endocytosis. This process involves the virus binding to cell surface receptors and undergoing pH-dependent conformational changes in the G1 glycoprotein, which facilitates membrane fusion and internalization (Hacker and Hardy 1997). Such molecular changes are critical for successful entry into host cells.

Another significant pathway CEV employs is the “Trojan Horse” in which infected macrophages or monocytes transport the virus across the BBB while evading immune surveillance (Al-Obaidi et al. 2018). CEV also directly infects endothelial cells lining blood vessels, leading to cell death and local inflammation. This localized damage further compromises BBB integrity, allowing easier viral penetration into the CNS. Once within the CNS, CEV triggers neuroinflammation that contributes to neuronal dysfunction and cell death. The host's immune response, while essential for clearing infection, can also exacerbate neural damage through inflammatory mediators (Wongchitrat et al. 2024). It has also been suggested that CEV may also utilize the olfactory nerves to directly access the CNS, bypassing the BBB entirely (Riel et al. 2015).

##### Neurological manifestations of CEV

Since its emergence in 1964, CEV has been an important cause of pediatric encephalitis in the United States, particularly in Midwestern states (Johnson et al. 1968). Infection typically develops with sudden fever, lethargy, and vomiting, followed by progressive signs of encephalitis over the first few days (Alissa et al. 2024; Sanghi et al. 2016). Most recover within one to two weeks, but seizures are a prominent symptom, occurring in roughly half of affected children Other neurological manifestations can include confusion, drowsiness, behavioral changes, or, in severe cases, coma (Siciliano et al. 2022). Some children also experience focal deficits such as involuntary muscle movements, weakness or paralysis, difficulties with speech, and poor coordination (Sanghi et al. 2016). Long-term consequences can occur, particularly in children who experienced seizures during the acute illness; up to one-fifth may develop recurrent, unprovoked seizures. Cognitive and memory deficits, persistent emotional changes, and epilepsy have also been reported in a subset of patients (Michaeli et al. 2014).

**Case studies/outbreaks of CEV**

**Table:** 

Year(s)	Location(s)	Reported cases	Neuroinvasive cases	Deaths	Notable features	References
1945	California, USA	3	3	3	Encephalitis	Young 1966
1966	California, USA	1	0	0	Blurry vision and dizziness	Eldridge et al. 2001

### Family reoviridae

#### Colorado tick fever virus (CTFV)

##### Mechanisms of CTFV CNS invasion

Colorado tick fever virus is transmitted to humans primarily through the bite of infected Rocky Mountain wood ticks, *Dermacentor andersoni*, which acquire the virus by feeding on infected small mammals such as squirrels, chipmunks, and mice (Harris et al. 2023). Humans become infected when bitten by these ticks, and while person-to-person transmission is rare, it can occur via blood transfusion or perinatal transmission (Harris et al. 2023; Gimenez-Richarte et al. 2022). CTFV is endemic to the western United States and western Canada, with cases reported in states such as Colorado, Wyoming, and California (Padgett et al. 2022).

Current evidence on the mechanisms by which CTFV enters the CNS is limited but growing. In vitro work has demonstrated that CTFV can replicate in human hematopoietic progenitors and persist within blood elements, particularly erythrocytes (Philipp et al. 1993). This ability to persist in circulating cells provides a potential pathway for hematogenous dissemination and suggests a “Trojan horse” model, in which infected cells may facilitate viral transit across the BBB. Direct interaction with endothelial cells represents a second experimentally supported route. In vitro studies have shown that CTFV can infect human endothelial cells and induce apoptosis, offering clear evidence that the virus can damage the endothelium (Owen et al. 2021). Such endothelial disruption could permit viral passage either directly through infected cells (transcellular transport) or indirectly by compromising barrier integrity and enabling paracellular leakage.

##### Neurological manifestations of CTFV

Although most CTFV infections present as a self-limited fever, headache, myalgia, and lethargy, with a biphasic illness pattern, neurological involvement has been documented in a minority of cases, particularly among children. While fatal cases are rare, up to 30% require hospitalization (McDonald et al. 2019). Reported CNS complications include aseptic meningitis, meningoencephalitis, and encephalitis, which may present with headache, photophobia, vomiting, and altered mental status (Kadkhoda et al. 2018; Prevention 2024). Seizures have been observed in pediatric patients, sometimes accompanied by irritability or lethargy, and can be an indicator of more severe disease progression. CTFV is frequently misdiagnosed as Rocky Mountain Spotted Fever due to similar initial presentations (Spruance and Bailey 1973). Neurological disease is generally acute, emerging within the first week of illness, and in most cases resolves without long-term sequelae. However, severe encephalitic presentations can occur, and rare cases of prolonged convalescence with cognitive or motor impairment have been reported (Prevention 2024). Pediatric patients appear more vulnerable to CNS involvement, consistent with observations in other arboviral encephalitides.

**Case studies/outbreaks of CTFV**

**Table:** 

Year(s)	Location(s)	Reported Cases	Neuroinvasive Cases	Deaths	Notable Features	References
1948–1959	Colorado, USA	115	7	1	All neurological complications in ages 2–10 years; encephalitis, meningeal irritation, meningoencephalitis	Spruance and Bailey 1973
1985–1995	USA	864	Not reported separately	0	Biphasic febrile illness common; isolated reports of encephalitis and meningitis	Padgett et al. 2022
2002–2022	USA	223	3	0	Documented neurological features include meningitis and encephalitis, mostly in pediatric and immunocompromised patients	Yendell et al. 2015; Fagre et al. 2024

### Emerging neurotropic threats

Although historically considered self-limiting febrile illnesses, in the last decade, sporadic reports of encephalitis and neuropathy caused by both Chikungunya and Mayaro viruses has transformed our understanding of these viruses from agents of mild febrile illness to potential neurotropic pathogens. This emerging neurotropic potential has drawn attention, highlighting the need to better understand their pathogenesis and clinical impact.

#### Chikungunya virus (CHIKV)

##### Mechanisms of CHIKV CNS invasion

Chikungunya virus is primarily transmitted by Aedes mosquitoes, particularly *Aedes aegypti* and *Aedes albopictus* that are highly adapted to urban and peri-urban environments (Sun et al. 2025). CHIKV is endemic in parts of Africa, Asia, and the Indian subcontinent, where sporadic and seasonal outbreaks occurw. In recent decades, the geographic range of CHIKV has expanded significantly, with outbreaks reported in the Americas and even parts of Europe, largely due to the global spread of competent mosquito vectors and increased human mobility (Bettis et al. 2022). Transmission intensity often peaks during rainy seasons when mosquito populations flourish, and both urban and rural cycles contribute to the persistence and spread of the virus (Lim et al. 2025).

The ability of CHIKV to invade the central nervous system CNS is a critical aspect of its pathogenesis, though the precise mechanisms remain incompletely understood. Some evidence indicates that CHIKV crosses CNS barriers through direct infection of endothelial or epithelial barrier cells and immune-cell–mediated transport, while axonal transport appears less prominent. Experimental studies, including in vivo imaging in zebrafish, show that CHIKV preferentially infects brain microvascular endothelial cells, supporting direct viral entry as a major route of neuroinvasion (Porto Silva et al. 2025). Infection of these barrier cells has been associated with vascular dysfunction and altered tight junction expression, increasing permeability and facilitating viral passage. Clinical-pathological studies of fatal cases further demonstrate disruption of the BBB, highlighting the potential for direct viral infection to weaken barrier defenses (Passoni et al. 2017).

Apart from direct infection of barrier cells, during acute infection, CHIKV can be detected in peripheral monocytes (de Souza et al. 2024; Brito et al. 2025). These infected monocytes are recruited toward the CNS in response to elevated chemokines, including CCL-2, and viral RNA has been identified within the CNS, providing evidence that monocytes transport CHIKV across the blood–brain barrier and contribute to neuroinflammation (Felipe et al. 2020).

Once within the CNS, CHIKV displays preferential tropism for glial and barrier-associated cells (Porto Silva et al. 2025). Astrocytes and other glial populations are readily infected and respond by upregulating pathogen-sensing pathways and pro-inflammatory gene expression (de Souza et al. 2024). In contrast, neurons and neural precursor cells, while less permissive in vivo, are susceptible to CHIKV infection in vitro and in animal models, where infection is associated with ultrastructural damage and increased apoptotic cell death (de Souza et al. 2024). This infection, together with the accompanying neuroinflammatory milieu, amplify CNS pathology. Pro-inflammatory cytokines such as IL-6, TNF-α, and type I interferons are elevated in the CSF and brain tissue of affected patients and experimental animals, contributing to further barrier disruption and activation of resident glial cells. Such neuroinflammation is thought to underlie clinical neurological manifestations and may have long-term consequences, although these effects remain to be fully characterized.

##### Neurological manifestation of CHIKV

CHIKV infection is primarily associated with arthralgic symptoms; however, it can also cause significant neurological complications, occurring in approximately 16.3% of infected patients (Porto Silva et al. 2025; de Souza et al. 2024). These neurological manifestations range from mild to severe and can affect individuals of various age groups, particularly those with pre-existing conditions. Commonly reported symptoms include headaches, which in adults may signal the onset of more serious complications (Chandak et al. 2009).

Clinically, severe cases often present as meningoencephalitis or, less frequently, as meningoencephalo-myeloradiculitis, reflecting combined involvement of the brain, spinal cord, and nerve roots. When classified anatomically, neurological involvement most commonly manifests as encephalitis (55%), followed by myelopathy (14%), peripheral neuropathy (14%), myeloneuropathy (14%), and rare cases of myopathy (2%) (Pinheiro et al. 2016; Almirón et al. 2024). GBS is another notable complication, reported in approximately 15% of patients with neurological symptoms, along with cognitive deficits in some cases (Chandak et al. 2009).

ADEM can also occur following CHIKV infection, mimicking other severe neurological disorders (Almirón et al. 2024; Brizzi 2017). The most severe cases may result in death, particularly among elderly patients with comorbidities, with some studies reporting mortality rates of up to 32% in patients with severe neurological manifestations (Alves-Leon et al. 2021).

**Case studies/outbreaks of CHIKV**

**Table:** 

Year(s)	Location(s)	Reported cases	Neuroinvasive cases	Deaths	Notable features	References
2005–2006	La Reunion Island	266,000	24	> 200	Encephalitis, encephalopathy and seizures	Bessaud et al. 2006; Almirón et al. 2024; Anand et al. 2019); Chastel et al. 2005; Bessaud et al. 2006
2006–2007	India	> 1.3million	Not reported separately	3,056	Altered mental functions, seizures, focal neurological deficits, optic neuritis and GBS reported	Kalantri et al. 2006; Chastel et al. 2005; Rampal et al., 2007; Kalantri et al. 2006
2014–2015	Brazil	> 3500	Not reported separately	6	Encephalitis, meningoencephalitis, acute myelitis, peripheral neuropathy	Health Brazil, Rampal et al. 2007

#### Mayaro virus (MAYV)

##### Mechanisms of MAYV CNS invasion

Mayaro virus is primarily transmitted by *Haemagogus janthinomys* and is considered endemic in parts of South America, with sporadic expansions into the Caribbean and Central America (Saúde 2016). Although classically described as an arthritogenic alphavirus, recent work has begun to clarify its potential to target the CNS (Wei et al. 2024).

In vitro studies provide the clearest foundation for MAYV’s neuroinvasive potential. The virus infects multiple components of the neurovascular unit including human neural progenitor cells, pericytes, astrocytes, and brain microvascular endothelial cells and these cell types consistently support productive viral replication (Bengue et al. 2021). Viral infection in these cells induces a pronounced antiviral and inflammatory program, characterized by the upregulation of interferon-stimulated genes and chemokines (Bengue et al. 2021). Such inflammatory activation is highly relevant to CNS access for both direct endothelial infection and virus-induced cytokine signaling that can weaken tight junctions, compromise endothelial barrier function, and promote increased BBB permeability. These observations align with broader patterns seen in other neurotropic arboviruses, in which BBB disruption during systemic infection provides a major gateway for CNS entry (Miner and Diamond 2016).

Beyond barrier breakdown, additional mechanisms may contribute to MAYV-CNS dissemination. A Trojan-horse model remains a plausible complementary pathway. Although direct mechanistic studies for MAYV are limited, clinical reports documenting MAYV RNA and lymphocytic pleocytosis in CSF suggest that infected monocytes or macrophages may carry the virus across the BBB. This leukocyte-mediated transit mirrors strategies used by several other encephalitic viruses and represents an important area for future experimental investigation.

Animal studies add further weight to the concept of neuroinvasion. Both mouse and non-human primate models have demonstrated the presence of MAYV RNA and infectious virus in CNS tissues and CSF, reinforcing the biological feasibility of CNS involvement and offering in vivo evidence that complements cell-based mechanistic data (Mutricy et al. 2022). These findings collectively highlight that MAYV, while not classically defined as a neurotropic alphavirus, possesses multiple pathways capable of enabling CNS access under the right immunological and inflammatory conditions.

##### Neurological manifestation of MAYV

Although neurological disease remains an infrequent outcome of MAYV infection, the combination of human case reports, CSF findings, and animal model data indicates that the virus is capable of producing clinically significant CNS and peripheral nervous system involvement under certain conditions. Reported manifestations range from mild and transient symptoms to more severe neuroinflammatory presentations (Weber et al. 2023). Patients may experience headache, photophobia, dizziness, and altered sensorium, often during the acute febrile phase (Silva And Krokovsky 2024). More pronounced complications such as meningitis, meningoencephalitis, and encephalitis have been documented in isolated human cases, typically accompanied by CSF abnormalities, most notably lymphocytic pleocytosis and detectable viral RNA.

Peripheral neurological symptoms, though rare, have also been reported. These include neuropathic pain, transient paresthesias, and occasional peripheral neuropathy, reflecting possible involvement of peripheral nerve pathways or immune-mediated mechanisms (Bengue et al. 2021; Mutricy et al. 2022). Experimental work in mouse and non-human primate models further supports CNS involvement, demonstrating viral RNA or infectious virus in brain tissue, microgliosis, and inflammatory changes consistent with viral neuroinvasion (Mutricy et al. 2022).

**Case studies/outbreaks of MAYV**

**Table:** 

Year(s)	Location(s)	Reported cases	Neuroinvasive cases	Deaths	Notable features	References
2003–2019	Various (Amazon and endemic regions)	About 17	1	0	Encephalitis with CSF pleocytosis; viral RNA detected in CSF	Mutricy et al. 2022; Bengue et al. 2021; Weber et al. 2023
2022	Brazil	1	1	0	Acute meningoencephalitis; neuroinvasion supported by molecular testing	Weber et al., 2023; Mutricy et al. 2022

## Prevention and therapeutic strategies

### Mosquito control strategies

The primary means of arboviral infection prevention is vector control. Older approaches, such as the spraying of larvicides to kill larvae and insecticides to kill adult mosquitoes, are still the most common (Berg et al. 2012). Newer approaches include the sterile insect technique that utilizes genetically modified sterile male mosquitos released into the environment around endemic areas to mate with females and produce no offspring (Pérez-Staples et al. 2021). Another involves the reduction of vector competence to harbor and transmit viral species, such as the *Wolbachia*-infected *Aedes aegypti* currently undergoing trials (Beebe et al. 2021; Pereira et al. 2018).

#### Vaccinations

Options exist for immunization-based prevention against arboviral infections, though only for a select few existing infections. The yellow fever vaccine is the oldest of these, in use since 1938. It consists of an attenuated virus with a very high (99%) rate of life-long protection 30 days after administration (Jean et al. 2016). In 2015, a DENV vaccine (Dengvaxia®, Sanofi) was approved for use in multiple countries of endemic nature. It was discontinued in some countries, especially in those without prior DENV exposure, for inducing deleterious immune responses. A second (QDENGA®, Takeda) was approved in 2022 in multiple countries. Dengue vaccination has proven to be difficult due to the need to avoid antibody-dependent enhancement of infection, thus a regimen was needed that induces a simultaneous response against all four serotypes (Lee et al. 2025). Other vaccines are available against the related viruses JEV and TBEV. Recently, a vaccine was approved for the alphavirus CHIKV (VIMKUNYA®, Bavarian Nordic) (Mudasir et al. 2026). Difficulties arise for this approach due to the need for a robust cold chain, leading to logistical issues in resource-poor settings. In addition, some individuals cannot receive vaccinations, especially those of the attenuated variety, instead often relying on high levels of community uptake. Continued development of arboviral immunizations is ongoing, with novel platforms such as VLPs (Ko et al. 2019) and MVA (Beddingfield et al. 2024) being of interest. Future vaccinations may provide for broader protection against a wider variety of viral species included within one regimen.

#### Therapeutics

No therapeutic interventions are currently available for arboviral infections. Multiple approaches are currently in development for various viral species, including monoclonal antibodies (mAbs) and small molecule antivirals. An antiviral often tested against viral species is the nucleoside analog ribavirin (Yeo et al. 2015), though its use is limited due to adverse events, especially when high doses are used (Knowles et al. 2003). Multiple known and novel antivirals are being evaluated for use in arboviral infections, including the polymerase inhibitor favipiravir (Furuta et al. 2013). Still other drugs are being tested that may aid in disease despite not functioning to inhibit viral replication directly, including immune modulators (Pryke et al. 2017; Rudd et al. 2012).

Monoclonal antibodies offer promise due to the very high therapeutic window and can sometimes function against multiple species of virus. Investigational mAbs include broadly neutralizing antibodies that inhibit receptor binding and internalization of virions (Sutton et al. 2023), as well as those inhibiting viral egress from the host cell (Williamson et al. 2021). Downsides of mAb-based therapy is that protection may be circumvented in the instance of viral evolution, as was seen during the recent SARS-CoV-2 pandemic (Jian et al. 2025), and have little potential to be repurposed beyond related species. Additionally, mAbs do not generally exhibit barrier crossing behavior the way that many small molecules do, thus limiting their usefulness after CNS entry of viral infections. This may be mitigated in the future with the use of single domain antibodies. These are variations of heavy-chain only camelid antibodies that possess binding and neutralization capability similar to antibodies but are significantly smaller and may cross barriers that inhibit traditional antibody therapeutics (Farrington et al. 2014; Soleimanizadeh et al. 2021). Other options may rely on intranasal delivery of full-length antibody, disruption of the BBB during drug delivery, or delivery via viral vector (Soleimanizadeh et al. 2021; Deverman et al. 2016; Kang et al. 2023).

## Conclusion

Arboviruses, and their associated infections, rely on multiple mechanisms to bypass physiological barriers, including the blood brain barrier. More work to elucidate these aspects of disease, especially with viral species not traditionally associated with CNS infections, remains a significant need.

## Data Availability

No datasets were generated or analysed during the current study.
